# Partial validation of a TaqMan quantitative polymerase chain reaction for the detection of the three genotypes of *Infectious spleen and kidney necrosis virus*

**DOI:** 10.1371/journal.pone.0281292

**Published:** 2023-02-03

**Authors:** Samantha A. Koda, Kuttichantran Subramaniam, Paul M. Hick, Evelyn Hall, Thomas B. Waltzek, Joy A. Becker

**Affiliations:** 1 Department of Infectious Diseases and Immunology, College of Veterinary Medicine, University of Florida, Gainesville, Florida, United States of America; 2 Emerging Pathogens Institute, University of Florida, Gainesville, Florida, United States of America; 3 Sydney School of Veterinary Science, The University of Sydney, Camden, New South Wales, Australia; 4 School of Life and Environmental Sciences, The University of Sydney, Camden, New South Wales, Australia; National Cheng Kung University, TAIWAN

## Abstract

Megalocytiviruses (MCVs) are double-stranded DNA viruses known to infect important freshwater and marine fish species in the aquaculture, food, and ornamental fish industries worldwide. *Infectious spleen and kidney necrosis virus* (ISKNV) is the type species within the genus *Megalocytivirus* that causes red seabream iridoviral disease (RSIVD) which is a reportable disease to the World Animal Health Organization (WOAH). To better control the transboundary spread of this virus and support WOAH reporting requirements, we developed and partially validated a TaqMan real-time qPCR assay (ISKNV104R) to detect all three genotypes of ISKNV, including the two genotypes that cause RSIVD. Parameters averaged across 48 experiments used a 10-fold dilution series of linearized plasmid DNA (10^7^–10^1^ copies), carrying a fragment of the three-spot gourami iridovirus (TSGIV) hypothetical protein revealed that the assay was linear over 7 orders of magnitude (10^7^–10^1^), a mean efficiency of 99.97 ± 2.92%, a mean correlation coefficient of 1.000 ± 0.001, and a limit of detection (analytical sensitivity) of ≤10 copies of TSGIV DNA. The diagnostic sensitivity and specificity for the ISKNV104R qPCR assay was evaluated and compared to other published assays using a panel of 397 samples from 21 source populations with different prevalence of ISKNV infection (0–100%). The diagnostic sensitivity and specificity for the ISKNV104R qPCR assay was 91.99% (87.28–95.6; 95% CI) and 89.8% (83.53–94.84). The latent class analysis showed that the ISKNV104R qPCR assay had similar diagnostic sensitivities and specificities with overlapping confidence limits compared to a second TaqMan qPCR assay and a SYBR green assay. This newly developed TaqMan assay represents a partially validated qPCR assay for the detection of the three genotypes of the species ISKNV. The ISKNV104R qPCR assay once fully validated, will serve as an improved diagnostic tool that can be used for ISKNV surveillance efforts and diagnosis in subclinical fish to prevent further spread of MCVs throughout the aquaculture and ornamental fish industries.

## Introduction

Aquaculture continues to be one of the fastest growing sectors of agriculture and reached a record high of an estimated $263 billion in 2018 [1]. Finfish production including inland, coastal, and marine production has dominated the aquaculture sector accounting for an estimated $140 billion annually [1]. With increasing economic importance and as a major global food source, the pressure on finfish production has also led to more frequent outbreaks of disease [2]. There has been an increasing concern that the exportation and importation of aquacultured fish are contributing to the spread of pathogens globally [3, 4]. The topic of transboundary movement of pathogens has not only been restricted to aquaculture and food fish but also to the global ornamental fish trade [5–9]. A key pathogen of global concern is the infectious spleen and kidney necrosis virus (ISKNV), as it is known to cause epidemics in numerous wild and farmed food and ornamental fish species.

ISKNV is known to cause disease in at least 165 different fish species across 53 fish families with the number of susceptible species continuing to increase in recent years [7, 10–22]. ISKNV has been detected across a wide range of countries (22) and continents (6) with a majority of the reports from Asia [8, 23–26]. In clinical cases of ISKNV infections, mortality can reach up to 100%, but most cases range from 20–60% mortality [6, 11, 27–38]. Clinical signs of disease are non-specific and include lethargy, gill pallor, skin discoloration, inappetence, and abnormal swimming. These gross clinical signs in addition to subclinical infections make surveillance efforts extremely important to control the spread of this virus.

The emergence of ISKNV infections in new host species and regions has led to a plethora of virus names to be given to isolates of the same virus species (e.g., see [39]) leading to inconsistent viral nomenclature. ISKNV is one of two classified species within the genus *Megalocytivirus* (MCV) [40]. Within the species ISKNV, there are three genotypes: RSIV (Genotype II), ISKNV (Genotype I), and turbot reddish body iridovirus (TRBIV) (Genotype III) [8]. These three genotypes can each be further subdivided into clades 1 and 2, for a total of six clades within the species ISKNV [8, 25, 41]. Henceforth, the term ISKNV will refer to the taxonomic level of species unless otherwise stated (e.g., ISKNV genotype). The three genotypes of ISKNV have overlapping host and geographic ranges. Noting its international importance, red seabream iridoviral disease (RSIVD), caused by infections with ISKNV and RSIV genotypes, is listed as one of 12 WOAH reportable fish diseases [42]. At the time of this publication, infection with TRBIV is not included in the definition of RSIVD, although this is being reviewed by WOAH Aquatic Animal Health Standards Commission (meeting held 14–21 September 2022). *Scale drop disease virus* is the second officially recognized species within the genus *Megalocytivirus* according to the International Committee on Taxonomy of Viruses (ICTV) [40]. SDDV first emerged as a pathogen of barramundi [36] and has recently caused epidemics in farmed Yellowfin Seabream (*Acanthopagrus latus*) [43]. In addition to SDDV, European chub iridovirus (ECIV) and three-spined stickleback (TSIV) are unclassified MCVs that have been proposed as species within the genus [44, 45].

To support global animal disease control, appropriate diagnostic tools that are fit for purpose and validated must be developed for WOAH reportable diseases like RSIVD. At present, several diagnostic tools have been developed for the detection of ISKNV including: conventional PCR [8, 46, 47], real-time PCR (qPCR; [48–52]), immunofluorescent antibody tests (IFAT) using a monoclonal antibody [53–55], virus isolation [56–61], DNA microarrays [62, 63], and loop-mediated isothermal amplification [64–66]. Out of all these diagnostic assays, there are only three recommended assays for RSIVD detection 1) a stamp smear showing megalocytic cell inclusions confirmed by an IFAT, 2) virus isolation with MCV-like characterized cytopathic effects (CPE) confirmed with an IFAT, and 3) a conventional PCR [67]. Of these, only the conventional PCR can differentiate between RSIV and ISKNV [67]. There is currently no validated assay to detect all three genotypes of ISKNV and this is a pressing need to develop a real-time quantitative polymerase chain reaction (qPCR) assay.

The development and validation of a highly sensitive and specific qPCR assay would be beneficial for the detection of ISKNV infections, especially with low viral loads as sometimes encountered during recent or resolving infections, and in subclinical carriers [68, 69]. The objective of this study was to develop and partially validate a TaqMan qPCR assay that detects all three ISKNV genotypes and here on out is referred to as the ISKNV104R qPCR assay. As part of the partial validation efforts, analytical and diagnostic sensitivity and specificity were evaluated for the ISKNV104R qPCR. Latent class analysis was performed to compare the diagnostic performance of the new assay with two previously published assays with different gene targets and/or assay chemistry [49, 70]. The intent of this work was to generate a qPCR assay useful in diagnostic laboratories with sufficient validation data for future inclusion as a WOAH acceptable test for surveillance and diagnosis of diseases caused by infections with ISKNV.

## Material and methods

### *In silico* TaqMan qPCR primer and probe design

Development and analytical performance of the ISKNV104R TaqMan qPCR were carried out at the University of Florida’s Wildlife and Aquatic Animal Veterinary Diagnostic Laboratory (WAVDL) in Gainesville, Florida, USA. An initial review of all published MCV genomes revealed that some MCVs (e.g., ECIV and SDDV) were too divergent from ISKNV strains (<75% amino acid identity), to be included in the scope of a single qPCR assay. Thus, ECIV and SDDV were not included in the sequence alignments used to generate the primers and TaqMan probe. Twelve fully sequenced genomes (S1 Table), representing all three ISKNV genotypes (ISKNV, RSIV, TRBIV), were downloaded from the National Center for Biotechnology Information (NCBI) GenBank database. A full genome alignment was performed using Mauve 2.4.0 with default settings [71]. From the output alignment, the locally collinear blocks (LCBs) were visually inspected for conserved sequence segments to be used to design the qPCR primers and probe. Once the LCBs were identified, the 100% consensus sequence for each segment was imported into Primer Express Software Version 3.0 (Applied Biosystems) to design an ISKNV species-specific primers and a [6FAM]-minor groove binder/non fluorescent quencher-labeled TaqMan hydrolysis probe using default settings. After the selection of suitable primers and a probe, an additional 11 fully sequenced MCVs were downloaded from GenBank to determine the *in silico* primer and probe specificity (S1 Table). All 23 genomes were aligned using MAFFT [72] and visualized in Bioedit v7.2.5 to inspect the specificity of the primers and probe [73].

### Generation of megalocytivirus DNA standards to assess assay performance

To test the efficiency of the new primers and probe, standard curves were evaluated from amplification of a linearized plasmid DNA standard that was generated through TOPO TA cloning. Additional primers were developed outside of the sequence to be amplified by the qPCR assay to generate an amplicon for cloning. A three spot gourami iridovirus sample (TSGIV; [8]) was used as the nucleic acid template for generating the target to be cloned. TSGIV was previously identified to belong to clade 2 of the TRBIV genotype (III) [8].

The volumes for the conventional PCR reactions were 30 μl and consisted of 0.15 μl of Platinum Taq DNA Polymerase (Invitrogen), 3.0 μl of 10× PCR Buffer, 1.2 μl of 50 mM MgCl_2_, 0.6 μl of 10 mM dNTPs, 1.5 μl of 20 μM of forward and reverse primers, 17.55 μl of molecular grade water, and 4.5 μl of DNA template. The reaction was carried out using a SimpliAmp thermal cycler (Applied Biosystems) using the following conditions: 94°C for 5 m, followed by 30 cycles at 94°C for 30 s, 56°C for 30 s, 72°C for 30 s, and final elongation at 72°C for 10 m. The presence of amplified products was confirmed by analyzing the PCR products on a 1% agarose gel stained with ethidium bromide. The expected band was purified using a QIAquick PCR Purification Kit (Qiagen). The concentration of the purified band was quantified using a Qubit® 3.0 Fluorometer and the dsDNA BR Assay Kit (Life Technologies). Once quantified, the purified DNA target was cloned into a vector (Invitrogen) and transformed into *E*. *coli* using the TOPO® TA Cloning® Kit (Invitrogen) following the manufacturer’s instructions. Transformed bacterial colonies were screened by conventional PCR using the M13 forward and reverse primers and the target was confirmed via Sanger sequencing. The colonies with the correct insert were grown in the LB broth overnight (16–20 hours) in an Incu-Shaker mini (Benchmark Scientific) at 37°C and 200 rpm. The following day, the tubes were centrifuged at 3000 x g for 10 minutes at 15°C before following the manufacturer’s protocol for plasmid purification using a QIAprep Spin Miniprep Kit (Qiagen).

The recombinant plasmid was linearized using the *NotI* enzyme (New England Biolabs) following the manufacturer’s instructions and analyzed on a 1% agarose gel stained with ethidium bromide. The correct size band containing the qPCR target was purified using a QIAquick PCR Purification Kit (Qiagen) and the DNA concentration was quantified as described above. The linearized plasmid was then diluted to 10^7^ copies/μl in TE buffer using the formula described by Krieg (1990) and aliquoted into 30 μl volumes and stored at -80°C until used. For each qPCR plate (termed experiment), an aliquot of 10^7^ copies of linear plasmid was thawed and serially diluted 10-fold with nuclease-free water using the QIAgility (Qiagen). Each standard dilution (10^7^–10^1^ copies) was run in triplicate (4 μl reaction well^-1^) to create the standard curve for each qPCR plate.

### Detection of the ISKNV DNA standard using the ISKNV104R qPCR assay

All qPCR plates were set up in a MicroAmp™ Fast Optical 96-Well Reaction Plate, 0.1 mL (Applied Biosystems) using the QIAgility (Qiagen). Each reaction contained 0.9 μM of each primer, 0.25 μM of probe, 4 μl of nucleic acid template (maximum of 50 ng of total DNA per reaction), 10 μl of universal qPCR mix (TaqMan® Fast Universal PCR Master Mix 2X, Applied Biosystems), and 3 μl of molecular grade water for a total volume of 20 μl. Once the plate was set up, it was sealed with a MicroAmp™ Optical Adhesive Film (Applied Biosystems), mixed on a 96 well plate shaker, and spun down before being evaluated by qPCR. The samples were run on a QuantStudio 5 Real-Time PCR System (Applied Biosystems) using the following fast qPCR protocol thermocycling conditions: 95°C for 20 s followed by 40 cycles at 95°C for 3 s and 60°C for 30 s. A threshold cycle (C_t_) value was automatically calculated by the QuantStudio^TM^ Design & Analysis Software v1.4.1 and interpreted as a positive result if the calculated C_t_ value as the proportional cycle number when the 6-carboxy-X-rhodamine (ROX)-normalized 6-carboxyfluorescein (FAM) signal exceeds the threshold assigned by the Applied Biosystems software. A sample was considered positive if 2/3 or 3/3 wells generated a C_t_ value ≤ 40.

Each plate included quality control samples including a negative extraction control, a no template control (molecular grade water), a standard curve (10^7^–10^1^), and a VetMAX™ Xeno™ Internal Positive Control (IPC) VIC Assay (Applied Biosystems). All the aforementioned samples and controls were run in triplicate wells with the ISKNV species-specific primers and probe except the VetMAX™ Xeno™ Internal Positive Control (IPC) which was added into the fourth well for each sample. The IPC reactions contained 0.8 μl of VetMAX™ Xeno™ Internal Positive Control–VIC™ Assay (Applied Biosystem), 1 μl of VetMAX™ Xeno™ Internal Positive Control DNA (Applied Biosystem) diluted 1:10, 4 μl of nucleic acid template, 10 μl of TaqMan™ Fast Universal PCR Master Mix (2X), and 4.2 μl of molecular grade water. IPC wells were deemed positive if the well generated a C_t_ value ≤ 35; indicating there was little to no PCR inhibition.

### Estimation of qPCR assay analytical sensitivity and specificity

Triplicate 10-fold dilutions of the TSGIV standard (10^7^−10^1^ copies) were used in each of 48 plates to estimate the correlation coefficient (R2), y-intercept, slope, efficiency, dynamic range, analytical sensitivity, repeatability, and reproducibility of the qPCR assay as previously described [74, 75]. Efficiency was calculated as 10-1/slope– 1 [76]. The qPCR assay limit of detection (LOD or analytical sensitivity) was defined as the lowest dilution at which 50% of positive samples (wells) were detected by a positive result. The coefficient of variation was used to assess intra-assay variability (repeatability) and inter-assay variability (reproducibility) for the 48 plates according to the formula CV% = [SD/mean] × 100. To estimate the qPCR analytical sensitivity further, infected tissues or isolates representing all three genotypes within the species ISKNV were used to determine the inclusivity of the assay for the three ISKNV genotypes (Table 1). Samples representing five of the six ISKNV clades (except clade 1 of the TRBIV genotype) were used to test the inclusivity of the ISKNV TaqMan qPCR. The ISKNV Clade 1, RSIV Clade 1, and TRBIV Clade 2 samples have been previously described by Koda et al. [77], Koda et al. [78], and Koda et al. [8], respectively. The genotype ISKNV clade 2 infected tissue DNA was extracted from the spleen and kidney from a Banggai cardinalfish *Pterapogon kauderni* that was confirmed by full genome sequencing (GenBank acc. no. MN432490). The genotype RSIV clade 2 infected tissue DNA was extracted from the liver of a diseased Florida pompano *Trachinotus carolinus* and was confirmed by PCR and Sanger sequencing using two MCV-specific primer sets [8].

**Table 1:** Summary of samples used in determining the analytical specificity and inclusivity of the ISKNV104R TaqMan qPCR assay.

Sample Name	Host Species (Common Name)	Host Species (Scientific Name)	Sample Type	Virus	MCV Clade	Reference
EFIV-2018	Albino rainbow shark	*Epalzeorhynchos frenatum*	Fresh kidney, spleen tissues	ISKNV	ISKNV clade 1	[77]
BCIV-2012	Banggai cardinalfish	*Pterapogon kauderni*	Fresh liver, kidney, spleen tissues	ISKNV	ISKNV clade 2	S.A. Koda (unpubl.)
PIV2010	Florida pompano	*Trachinotus carolinus*	Frozen kidney, spleen tissues	ISKNV	RSIV clade 1	[78]
WVL17011	Florida pompano	*Trachinotus carolinus*	Frozen liver tissue	ISKNV	RSIV clade 2	T.B. Waltzek (unpubl.)
TSGIV	Three spot gourami	*Trichopodus trichopterus*	Viral isolate	ISKNV	TRBIV clade 2	[8]
LEC15001	European chub	*Squalius cephalus*	Viral isolate	European chub iridovirus		[45]
SA15064	Pallid sturgeon	*Scaphirhynchus albus*	Viral isolate	Frog virus 3		[80]
FOF15001	Longnose butterflyfish	*Forcipiger flavissimus*	Frozen fin tissue	Lymphocystivirus		[79]
WVL21010	Koi carp	*Cyprinus carpio*	Frozen skin tissue	Carp edema virus		[82]
CCK14138D	Koi carp	*Cyprinus carpio*	Fresh skin tissue	Cyprinid herpesvirus 1		[81]
CARA14004A	Goldfish	*Carassius auratus*	Fresh skin tissue	Cyprinid herpesvirus 2		[81]
CCK14139D	Koi carp	*Cyprinus carpio*	Fresh skin tissue	Cyprinid herpesvirus 3		[81]

To estimate the qPCR analytical specificity, the assay was tested against DNA from an isolate of a divergent, unclassified MCV, European chub iridovirus (ECIV; [45], as well as other previously described tissues infected with iridoviruses of the closely related genera, *Lymphocysitivirus* [79] and *Ranavirus* [80]. Other double-stranded DNA viruses including cypriniviruses (*Cyprinid herpesvirus 1*–*3*, CyHV1-3) [81] and carp edema virus (CEV) [82] were also tested against the ISKNV TaqMan qPCR assay (Table 1).

### Estimation of qPCR assay diagnostic sensitivity and specificity for 3 assays

The diagnostic performance of the ISKNV104R TaqMan qPCR assay was evaluated at The University of Sydney, Camden, NSW, Australia. The fish tissues samples used were approved by The University of Sydney Animal Ethics Committee (2014/720; 2016/979). A panel of 397 samples of pooled liver, kidney, and spleen tissue homogenates was acquired from a cross sectional surveillance survey and challenge studies that were performed at The University of Sydney (Table 2). In total, the 397 samples represented 21 different fish populations to test the diagnostic performance. Apparent prevalence in these populations was estimated by prior testing (using [49]) and populations were categorized according to low (<10%), medium (10–70%), and high prevalence (>70%). For the low prevalence populations, we selected all the positive samples in the low prevalence populations and then randomly selected negatives from the same population. For the medium and high prevalence populations, we randomly selected positives and purposively selected negatives. Diagnostic sensitivity was assessed based on all 21 populations, while three populations that were free from infection and represented true negative samples (Populations 15–17; 36 samples) were used to assess diagnostic specificity. The panel of validation samples from surveillance in Australia included a range of samples (Table 2) from those with low viral load (< 100 viral genome copies mg^-1^ tissue) and low prevalence (<10% of the population) to samples with high viral load (8.6 x 10^9^ viral genome copies mg^-1^ tissue) and 100% population prevalence. All sample populations were determined to be the genotype ISKNV by testing using the SYBR green assay [49] and genotyping via three conventional PCRs followed by Sanger sequencing [83, 84].

**Table 2:** Summary of fish population samples used to determine the diagnostic sensitivity and specificity for comparison of the ISKNV104R, RSIV RT, and C1073/C1074 qPCR assays.

Population ID	Sample Description	Collection Date	Exporting Country	Host Species	Apparent Prevalence (%)	Number of Samples Selected	
					Positive^a^	Negative^a^	
1	Surveillance	January 2015	Indonesia	Five-lined cardinalfish *Cheilodipterus quinquelineatus*	25 –medium	8	7
2	Surveillance	January 2015	Indonesia	Blue green damselfish *Chromis viridis*	2 –low	0	2
3	Surveillance	January 2015	Indonesia	Five-lined cardinalfish *Cheilodipterus quinquelineatus*	32 –medium	13	2
4	Surveillance	May 2015	Singapore	Southern platyfish *Xiphophorus maculatus*	7 –low	5	5
5	Surveillance	May 2015	Thailand	Three spot gourami *Trichopodus trichopterus*	85 –high	10	5
6	Surveillance	May 2015	Thailand	Southern platyfish *Xiphophorus maculatus*	5 –low	4	2
7^b^	Surveillance	May 2015	Singapore	Three spot gourami *Trichopodus trichopterus*	4 –low	7	2
8	Surveillance	October 2015	Malaysia	Three spot gourami *Trichopodus trichopterus*	33 –medium	22	10
9	Surveillance	October 2015	Malaysia	Green swordtail *Xiphophorus helleri*	65 –medium	40	21
10	Surveillance	October 2015	Malaysia	Freshwater angelfish *Pterophyllum scalare*	30 –medium	17	10
11	Surveillance	October 2015	Malaysia	Dwarf gourami *Trichogaster lalius*	11 –medium	10	6
12	Surveillance	October 2015	Sri Lanka	Green swordtail *Xiphophorus helleri*	39 –medium	26	11
13	Surveillance	October 2015	Sri Lanka	Southern playtfish *Xiphophorus maculatus*	79 –high	45	29
14	Surveillance	October 2015	Sri Lanka	Three spot gourami *Trichopodus trichopterus*	46 –medium	13	7
15	Experimental trial–not exposed	June 2017	N/A	Murray cod *Maccullochella peelii peelii*	0	0	4
16	Experimental trial–not exposed	August 2017	N/A	Murray cod *Maccullochella peelii peelii*	0	0	9
17	Experimental trial–not exposed	November 2017	N/A	Murray cod *Maccullochella peelii peelii*	0	0	23
18	Experimental trial–exposed	August 2017	N/A	Murray cod *Maccullochella peelii peelii*	90 –high	7	3
19	Experimental trial–exposed	August 2017	N/A	Murray cod *Maccullochella peelii peelii*	92 –high	4	1
20	Experimental trial–exposed	November 2017	N/A	Murray cod *Maccullochella peelii peelii*	56 –medium	0	4
21	Experimental trial–exposed	November 2017	N/A	Murray cod *Maccullochella peelii peelii*	100 –high	3	0

^a^Determined by Rimmer et al. 2012

^b^Genotype could not be determined and was assumed to be ISKNV as fish received on the same from the same supplier were confirmed to be ISKNV (Population 4).

The diagnostic performance of the newly developed ISKNV104R qPCR assay was performed at the University of Sydney and was assessed by testing the samples in duplicate 25 μl reactions prepared with TaqMan™ Universal Master Mix (ThermoFisher). Reactions were prepared with 12.5 μl mastermix with 0.6 μM of each primer, 0.25 μM of probe, 2 μl of nucleic acid template, and nuclease free water. The reaction mixtures were loaded in a 96-well plate sealed with optically clear strip caps (Agilent). Each plate included duplicate no-template controls (nuclease free water), negative extraction control, and a PCR positive ISKNV genomic.

DNA sample, all tested in duplicate. Reactions were carried out on a Mx3000P qPCR system (Stratagene) using the following thermal cycling conditions: 95°C for 10 m, followed by 45 cycles at 95°C for 15 s and 60°C for 60 s. The sample was interpreted as a positive result if the ROX-normalized FAM signal exceeded the threshold assigned by the MxPro software (Stratagene) in at least one of the duplicate reaction wells.

Partial validation of the newly developed ISKNV104R qPCR assay was conducted with the operator blinded to the sample status to test the panel. A second TaqMan qPCR assay (RSIV RT) using the primers RSIV RT F/R and RSIV probe [70] was conducted consistent with the Australian and New Zealand Standard Diagnostic Procedure [85] and a SYBR green qPCR assay (C1073/C1074) using the C1073 and C1074 primers [49]. Each assay used aliquots of the nucleic acids at the same number of freeze thaw cycles as the ISKNV104R TaqMan qPCR assay. Similarly, all reactions (including no template controls) were run in duplicate in 96-well 0.2 ml qPCR plates sealed with optically clear strip caps (Agilent) on a Mx3000P qPCR system (Stratagene). The qPCR assay reactions for the RSIV RT TaqMan assay [70] contained 12.5 μl TaqMan™ Universal Master Mix (Thermofisher), 0.9 μM of each primer, 0.25 μM of probe, and 2 μl of nucleic acid template with nuclease free water to a total reaction volume of 25 μl. The following thermocycling conditions were used for the RSIV RT assay: 50°C for 2 m, 95°C for 10 m, and 45 cycles of 95°C for 15 s and 60°C for 60 s. The qPCR assay reactions for the C1073/C1074 SYBR green assay [49] contained 12.5 μl QuantiTect SYBR Green Master Mix (Qiagen), 0.5 μM of each primer, 5 μl of nucleic acid template and nuclease free water for a total reaction volume of 25 μl. The C1073/C1074 assay was run using the thermal cycling conditions: 95°C for 15 m, followed by 40 cycles of 95°C for 30 s, 62°C for 30 s, and 72°C for 30 s followed by a melting curve. All PCR plates included duplicate reactions prepared with a no template control, negative extraction control and two positive controls containing ISKNV or RSIV genomic DNA. The appropriate plasmid DNA standard (MCP or ISKNV104R) was amplified (10^7^−10^2^ copies/reaction) on each plate to determine absolute quantification. The fluorescence threshold was set for each plate based on amplification of the standard and applied to the experimental samples to assign a C_t_ value or negative result. The samples were assessed for inhibition using a VetMax Xeno Internal Positive Control assay (Thermofisher). A positive sample for the TaqMan assays were defined as having at least one of two duplicate wells amplifying. A positive result for the SYBR green assay was defined as having a melting temperature of 85.0 ± 0.5°C.

A Bayesian approach to latent class analysis was used to analyze the diagnostic sensitivity and specificity of the three qPCR assays. It was assumed that the three tests were independent due to their different chemistry and/or different target genes for amplification. WinBUGS (Version 1.4.3, 2007) was used to run the model for 100,000 iterations, discarding the first 10,000.

## Results

### *In silico* TaqMan qPCR primer and probe design

**Fig 1:**
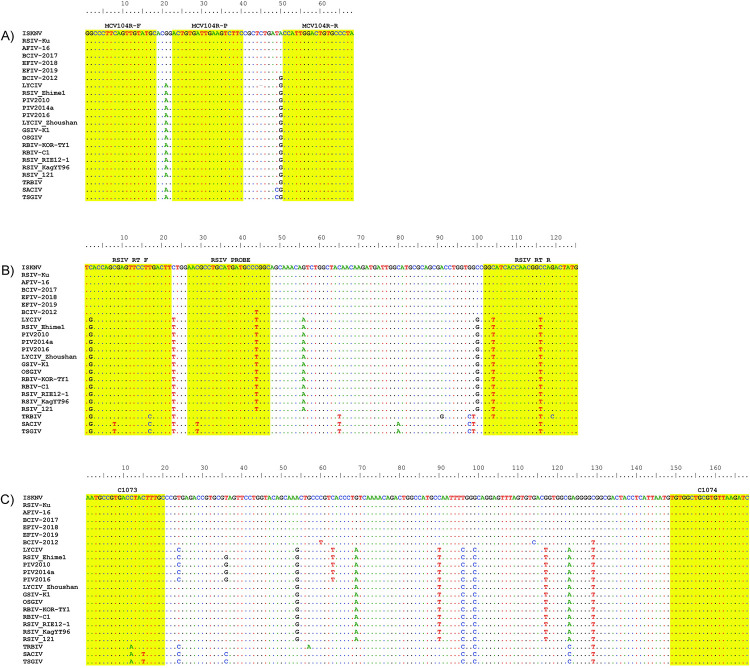
Aligned partial (68 bp) hypothetical protein ISKNV 104R (A) for 23 megalocytivirus strains illustrating the *in silico* specificity of the qPCR primers and probe of ISKNV TaqMan qPCR assay (this study). Aligned partial major capsid protein (MCP) for the same 23 megalocytivirus strains illustrating the *in silico* specificity of the qPCR primers RSIV RT F/R and RSIV probe (B; [69]) and C1073/C1074 (C; [48]).

Visual inspection and *in silico* analyses of the resulting consensus sequences led to the identification of ISKNV ORF104R (GenBank Acc. No. AF371960), which encodes a hypothetical protein, as a suitable target for this ISKNV TaqMan qPCR assay. The primer (ISKNV104R-F and ISKNV104R-R) and probe (ISKNV104R-P) sequences that were selected for the TaqMan qPCR assay targeted a 68 bp region for amplification of the hypothetical protein mentioned above (Table 3). *In silico* analysis of the ISKNV104R primers and probe revealed no mismatches to the original 12 MCV genomes that were used to develop the assay (Fig 1). Similarly, no mismatches were found after aligning the novel ISKNV104R primers/probe to an additional 11 ISKNV genomes, encompassing all 6 MCV clades within the species ISKNV, that subsequently became available in NCBI GenBank.

**Table 3:** Primers and probe sequences used in the TaqMan ISKNV104R qPCR targeting the hypothetical protein (ISKNV ORF104R GenBank acc. no. AF371960).

Primer/probe name	Sequence (5’−3’)	Melting temp. (°C)	Amplicon size (nt) including primers
ISKNV104R-F	GGCCCTTCAGTTGTATGC	59.9	68
ISKNV104R-R	TAGGGCACAGTCCAATGG	59.9	
ISKNV104R-P	ACTGTGATTGAAGTCTTC		

### Estimation of qPCR assay analytic sensitivity and specificity

**Fig 2:**
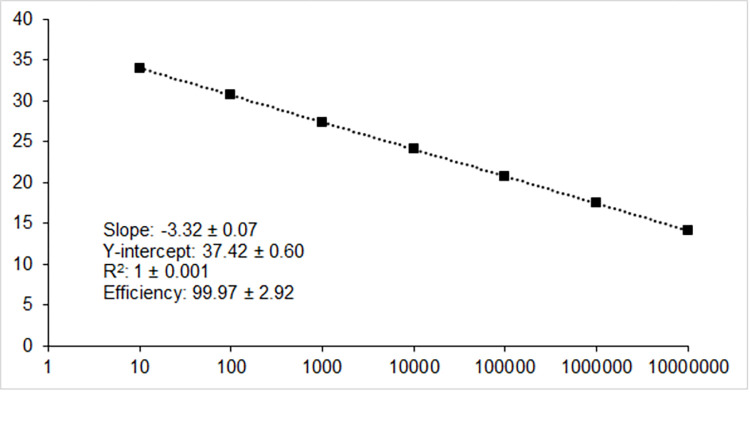
TaqMan ISKNV 104R qPCR assay standard curve generated using triplicate 10-fold serial dilutions of a MCV standard ranging from 10^7^-10^1^copies. The mean qPCR assay parameters (± SD) averaged over the 48 experiments are provided. The x-axis shows the standard copy number, and the y-axis indicates the corresponding cycle threshold.

A 10-fold dilution series of linearized plasmid DNA (10^7^–10^1^ copies), carrying a 346 bp fragment of the TSGIV hypothetical protein (GenBank acc. no. MG570132, positions 91979–92755) was used for the standard curves. The amplification plot revealed that the qPCR assay was linear over 7 orders of magnitude (10^7^–10^1^) (Fig 2). The mean parameters (± SD) for the qPCR assay averaged over the 48 experiments were as follows: slope = -3.32 ± 0.07, y-intercept: 37.42 ± 0.60, R^2^: 1 ± 0.001, and efficiency: 99.97 ± 2.92% (Fig 2). The LOD of the assay (analytical sensitivity) was determined to be 10 copies of ISKNV DNA standard per reaction (positive in 144/144 of the reactions with 10 copies of template; Table 4). The coefficient of variation of intra- and inter-assay C_t_ values ranged from 0.1–2.25% and 0.15–1.55%, respectively (Table 4).

**Table 4:** Inter-assay (reproducibility) and intra-assay (repeatability) variability of the TaqMan ISKNV104R qPCR assay. To determine reproducibility, the reactions for each standard (10^7^−10^1^ copies) were run in triplicate across 48 experiments. To determine repeatability, data obtained in a single representative qPCR run using standard (10^7^−10^1^copies) in triplicate. C_t_: threshold cycle number; CV: coefficient of variation; SD: standard deviation.

Standard dilution	Inter-assay reproducibility				Intra-assay repeatability			
			CV (%)	No. of positive wells (n = 144)			CV (%)	No. of positive wells (n = 3)
	C_t_ Mean	C_t_ SD			C_t_ Mean	C_t_ SD		
10^7^	14.176	0.09	0.60	144	14.443	0.08	0.57	3
10^6^	17.460	0.06	0.34	144	17.765	0.05	0.31	3
10^5^	20.757	0.06	0.30	144	21.026	0.08	0.36	3
10^4^	24.095	0.04	0.15	144	24.329	0.02	0.10	3
10^3^	27.433	0.08	0.29	144	27.631	0.11	0.41	3
10^2^	30.779	0.13	0.43	144	30.944	0.12	0.40	3
10^1^	33.986	0.53	1.55	144	34.489	0.78	2.25	3

The five samples representing five of six of the ISKNV clades tested positive. For analytical specificity, all samples tested negative. The VetMAX™ Xeno™ internal positive controls tested positive for all samples, indicating that the reactions were not inhibited by any of the substances in the samples.

### Estimation of qPCR assay diagnostic performance and latent class analysis

Using the prior results and classification of the C1073/1074 assay [49] for the 397 panel of samples, the diagnostic sensitivity and specificity for the ISKNV104R qPCR was found to be 91.99% (95% Confidence Interval (CI): 87.28–95.6%) and 89.81% (95% CI: 83.53–94.84%), respectively (Table 5). The latent class analysis revealed that the C1073/C1074 assay had the highest diagnostic sensitivity at 93.54% (95% CI: 88.86–97.06%) while the RSIV RT assay had the lowest diagnostic sensitivity at 87.8% (95% CI: 82.35–92.22%). For diagnostic specificity, all three assays performed about the same with the RSIV RT assay performing slightly better than the other two at 91.83% (95% CI: 86.2–96.02%). The C1073/C1074 SYBR green assay was the second best performing at 90.71% (95% CI: 84.78–95.35%), followed by the ISKNV104R qPCR assay with 89.81% (95% CI: 83.53–94.84%).

**Table 5:** Summary of the characteristics and predicted diagnostic performance of the ISKNV TaqMan qPCR (this study), RSIV TaqMan qPCR, and ISKNV SYBR Green qPCR assays using a Bayesian latent class analysis with a 95% confidence interval (CI).

Assay Name	Assay Type	Target Gene	Sensitivity (95% CI)	Specificity (95% CI)	Youden Index	Limit of Detection
ISKNV104R (This study)	TaqMan	ISKNV ORF104R	91.99% (87.28–95.6)	89.8% (83.53–94.84)	0.818	≤10 copies
RSIV RT (Mohr et al. 2015)	TaqMan	MCP	87.8% (82.35–92.22)	91.83% (86.2–96.02)	0.7963	Not reported
C1073/C1074 (Rimmer et al. 2012)	SYBR Green	MCP	93.54% (88.86–97.06)	90.71% (84.78–95.35)	0.8425	100 copies

MCP: major capsid protein

## Discussion

As a highly virulent virus in fish, ISKNV infections continue to be reported in new geographical regions around the world due to increased awareness, surveillance, and disease outbreaks [21, 77, 86, 87]. Since the first published report of RSIV in 1990 from red seabream, several diagnostic assays have been developed to identify ISKNV species [88]. The importance of a properly validated diagnostic tool became apparent when RSIVD was classified as an WOAH reportable disease in 2000 [67]. However, as outlined in the WOAH Manual of Diagnostic Tests for Aquatic Animals [67], only three assays have been partially validated for the detection of genotypes RSIV and ISKNV and none have been formally validated as outlined in the WOAH chapter Principles and Methods of Validation of Diagnostic Assays for Infectious Diseases [89]. At present, only two real-time PCR assays have been partially validated with consideration of all three ISKNV genotypes which presents a major gap in detecting this pathogen and preventing the pathogen from spreading further globally [90, 91]. The lack of a fully validated qPCR assay highlights the need for an assay that is applicable to surveillance and diagnosis of diseases caused by each of the ISKNV genotypes [49, 68, 70, 92]. The ISKNV104R qPCR assay reported here was optimized and partially validated to Stage 2 [89] to detect all 3 genotypes of the species ISKNV. This assay is further differentiated by testing against samples representing five of the six clades within the species ISKNV. After the completion of Stage 2 validation, a diagnostic assay can be given the designation of ‘provisional recognition’ and this will support the progression to Stage 3 evaluation and classification as being ‘validated for the original intended purposes’ [89].

The analytic performance of the ISKNV104R qPCR assay exhibited high coefficient of correlation, efficiency, sensitivity (LOD, was less than 10 copies reaction^-1^), specificity (100%), repeatability, and reproducibility. Recently, a TaqMan qPCR assay was developed targeting the RSIV major capsid protein and was partially validated with an even lower LOD of 2.5 viral genome copies [91]. Validation of diagnostic assays with extremely low limits of detections are important in subclinical cases where the animal may have early or recovering infection. These cases will be of utmost importance in surveillance and preventing the spread of the virus globally. In comparison to previous partially validated qPCR assays, other analytical sensitivity parameters (correlation coefficient and efficiency) of the ISKNV104R qPCR assay were found to be one of the highest resulting in an overall R^2^ value of 1 ± 0.001 and an efficiency of 99.97% ± 2.92% [49, 90, 91, 93]. A previous study however by Johnson et al. (2019) found that the analytical sensitivity of the C1073/C1074 SYBR green assay was reduced by two orders of magnitude when the plasmid was spiked into fish tissues compared to molecular grade water. These results suggest that there is some level of inhibition that is carried through the DNA extraction process and may result in decreased detection of the pathogen. So, while high analytical sensitivity is a good characteristic of a diagnostic tool, it is not truly representative of a tissue sample since the plasmid is in a matrix of molecular grade water. Analytical specificity was also found to be very high with no amplification of any of the divergent MCVs, related iridovirus genera, cypriniviruses, or CEV.

One of the main criteria for validation of a diagnostic assay is that it must be validated for the species for which the assay will be used for [89]. However, this would be very difficult given ISKNV has been detected in >160 species of fish [94]. In testing the analytical and diagnostic specificity of the ISKNV104R qPCR assay, DNA tissue samples from 397 individuals from 14 fish species were used to provide evidence that the assay is robust. Additionally, in comparison to other ISKNV qPCR studies [90, 91], the diagnostic performance of ISKNV104R qPCR assay was determined using the largest number of tissue samples and greatest range of host species. The samples that were used to test the analytical and diagnostic performance were also unique in that we used both freshwater and marine fish species which has not been done in previous qPCR studies. Moreover, DNA was extracted from different types of samples including fresh tissues, frozen tissues, and viral isolates which represent samples typically received by diagnostic laboratories. The usage of different types of samples helped prove the high performance of analytical selectivity with this assay and may provide a more realistic outlook when tested at the reproducibility and implementation stage of WOAH validation pathway. In testing for diagnostic performance of the three qPCR assays, the use of latent class analysis using Bayesian estimation was instrumental in making the comparison between the three [95]. This requires the assumption that qPCR assays with different detection chemistries and amplification targets are independent tests. In conclusion, all three assays performed relatively equally despite using different types of samples across 14 different fish species. The validation data can be used by regulator authorities and diagnostic laboratories involved in testing to support biosecurity for the international trade in fish and fish products.

The TaqMan ISKNV104R qPCR assay developed in this study represents the first qPCR assays to test five out of six ISKNV clades. The only ISKNV clade that was not tested due to limited access to samples was TRBIV Clade 1. However, *in silico* analyses have predicted that this assay is able to detect all six ISKNV clades (Fig 1). The genotype TRBIV has been excluded as a causative agent in RSIVD despite having high genetic similarity to the other two genotypes. Consequently, the WOAH Manual of Diagnostic Tests for Aquatic Animals [67] does not include validation against TRBIV when diagnostic assays for RSIVD were originally recommended over 20 years ago. A review of the literature shows that the genotype TRBIV was initially only reported in Asian flatfish species and has been reported less frequently in comparison to the genotypes ISKNV and RSIV which may be a reason for its exclusion [21, 39, 96–101]. However, there was a recent TRBIV outbreak in 2017 reported from Taiwan in a popular food fish, barramundi *Lates calcarifer* [102]. Previously, TRBIV has also been confirmed by PCR in rock bream (*Oplegnathus fasciatus*) from Taiwan, another popular marine food fish species in Asia [103]. The WOAH Aquatic Animal Health Standards Commission is undertaking a review of the chapter addressing RSIVD with the consideration of including the TRBIV genotype in the definition of infection. If TRBIV is included, the partially validated pan-ISKNV assay developed herein could be considered for inclusion in the WOAH manual [67] and this would support future work to further validate the assay. The purpose of the TaqMan ISKNV104R qPCR assay was to develop and partially validate the first qPCR diagnostic tool for the intended purpose of ISKNV surveillance. Particularly, to certify freedom from infection or presence of the agent in individual animals and in a defined population in trade/movement as outlined in the WOAH validation of diagnostic assays for infectious diseases [89].

The reported ISKNV104R qPCR assay provides a diagnostic tool that is cost-effective, rapid, highly specific and sensitive, and quantitative in comparison to the other diagnostic tools such as conventional PCR and virus isolation. Analysis of the *in silico* primer and probe mapping to 23 MCVs representing all six MCV clades resulted in zero mismatches. Analytical and diagnostic performance of the TaqMan ISKNV104R qPCR assay was found to be high and comparable to that of the SYBR green C1073/C1074 and TaqMan RSIV RT. With the number of fish species reported to be susceptible to ISKNV infections continuing to increase, the need for validated qPCR assays for surveillance and diagnosis is extremely important. Stocks of many important food fish species such as Nile tilapia (*Oreochromis niloticus*), barramundi, red sea bream (*Pagrus major*), rock bream, and grouper species (*Epinephelus* spp.) have experienced significant mortality events due to ISKNV [10, 15–18, 37, 38, 87, 101, 104–106]. This is key for the aquaculture industry where there has been a continual increase in production which has also resulted in more frequent pathogen outbreaks [2]. Co-infections with two different ISKNV genotypes have also been reported in the literature and serve as another justification for the development of a pan-ISKNV assay [107, 108]. Thus, the ability to detect all ISKNV genotypes will be an important feature of any diagnostic tool used in global ISKNV surveillance efforts to ultimately prevent the spread of these pathogens. Additional efforts are needed to test the reported ISKNV TaqMan qPCR against other sample types including DNA derived from ISKNV isolates, RSIV samples, and TRBIV samples. Lastly, the next steps for validation of this assay would include testing this assay with the same panel of samples plus WOAH reference samples at multiple laboratories to determine reproducibility, and finally implementation.

## Supporting information

S1 Table Genome summary of the 23 megalocytivirus genomes used to develop and/or demonstrate the *in silico* specificity of the ISKNV TaqMan qPCR primers (ISKNV104R-F and ISKNV104R-R) and TaqMan probe (ISKNV104R-P). (DOCX)

S2 Table Raw data used to determine the standard curve for the ISKNV 104R qPCR assay with the mean and standard deviation (SD) calculated for the 48 experiments at each serial dilution (10^7 to 10^1). (XLSX)
