# Supplementary material for: Partial validation of a TaqMan quantitative polymerase chain reaction for the detection of the three genotypes of Infectious spleen and kidney necrosis virus
Source: PLoS One. 2023 Feb 3;18(2):e0281292. doi: 10.1371/journal.pone.0281292 (PMC9897559; doi:10.1371/journal.pone.0281292)
Supplement: S1 Table — (DOCX) [file pone.0281292.s001.docx]

**S1 Table. Genome summary of the 23 megalocytivirus genomes used to develop and/or demonstrate the *in silico* specificity of the ISKNV TaqMan qPCR primers (ISKNV104R-F and ISKNV104R-R) and TaqMan probe (ISKNV104R-P).**

| Strain Name | Host Species | GenBank Acc. No. | Genome Size (bp) | Reference | | | |
| --- | --- | --- | --- | --- | --- | --- | --- |
| AFIV-16 | Freshwater angelfish  *Pterophyllum scalare* | MK689685 | 111,127 | Kawato et al. 2020 |  |  |  |
| BCIV-2012 | Banggai cardinalfish  *Pterapogon kauderni* | MN432490 | 112,618 | Koda et al. unpubl. |  |  |  |
| BCIV-2017 | Banggai cardinalfish  *Pterapogon kauderni* | MT926123 | 111,920 | Koda et al. unpubl. |  |  |  |
| EFIV-2018 | Albino rainbow shark  *Epalzeorhynchos frenatum* | MW273353 | 111,369 | Koda et al. 2021 |  |  |  |
| EFIV-2019 | Albino rainbow shark  *Epalzeorhynchos frenatum* | MW273354 | 111,380 | Koda et al. 2021 |  |  |  |
| GSIV-K1^a^ | Barramundi  *Lates calcarifer* | KT804738 | 112,565 | Wen & Hong 2016 |  |  |  |
| ISKNV^a^ | Mandarinfish  *Siniperca chuatsi* | AF371960 | 111,362 | He et al. 2007 |  |  |  |
| LYCIV^a^ | Yellow croaker  *Larimichthys crocea* | AY779031 | 111,760 | Ao & Chen 2006 |  |  |  |
| LYCIV_Zhoushan | Yellow croaker  *Larimichthys crocea* | MW139932 | 112,043 | Wang et al. unpubl. |  |  |  |
| OSGIV^a^ | Orange-spotted grouper  *Epinephelus coioides* | AY894343 | 112,636 | Lu et al. 2005 |  |  |  |
| PIV-2010 | Florida pompano  *Trachinotus carolinus* | MK098185 | 112,321 | Koda et al. 2018 |  |  |  |
| PIV-2014a | Florida pompano  *Trachinotus carolinus* | MK098186 | 112,377 | Koda et al. 2018 |  |  |  |
| PIV-2016 | Florida pompano  *Trachinotus carolinus* | MK098187 | 112,052 | Koda et al. 2018 |  |  |  |
| RBIV-C1^a^ | Barred knifejaw  *Oplegnathus fasciatus* | KC244182 | 112,333 | Zhang et al. 2014 |  |  |  |
| RBIV-KOR-TY1^a^ | Red seabream  *Pagrus major* | AY532606 | 112,080 | Do et al. 2004 |  |  |  |
| RSIV Ehime-1^a^ | Red seabream  *Pagrus major* | AB104413 | 112,415 | Kurita et al. 2002 | |  |  |
| RSIV KagYT-96 | Red seabream  *Pagrus major* | MK689686 | 112,710 | Kawato et al. 2020 | |  |  |
| RSIV RIE12-1^a^ | Red seabream  *Pagrus major* | AP017456 | 112,590 | Matsuyama et al. 2017 | |  |  |
| RSIV_121 | Barramundi  *Lates calcarifer* | MT798582 | 111,557 | Puneeth et al. unpubl. | |  |  |
| RSIV-Ku^a^ | Red seabream  *Pagrus major* | KT781098 | 111,154 | Wen & Hong 2015 | |  |  |
| SACIV^a^ | Keyhole cichlid  *Cleithracara maronii* | MG570131 | 111,347 | Koda et al. 2018 | |  |  |
| TRBIV^a^ | Turbot  *Scophthalmus maximus* | GQ273492 | 110,104 | Shi et al. 2010 | |  |  |
| TSGIV^a^ | Threespot gourami  *Trichopodus trichopterus* | MG570132 | 111,591 | Koda et al. 2018 | | |  |

^a^These genomes were used in the initial MAFFT alignment for the development of the MCV qPCR assay
